# Dataset on the expansion and consolidation of flooded settlements in the Dosso Region, Niger

**DOI:** 10.1016/j.dib.2022.107935

**Published:** 2022-02-10

**Authors:** Maurizio Tiepolo, Andrea Galligari

**Affiliations:** Interuniversity Department of Regional and Urban Studies and Planning, Politecnico and University of Turin, Viale Mattioli 39, Turin 10125, Italy

**Keywords:** Built-up area, Disaster risk reduction, Flood damage, Land cover, Niger, Rural settlements, Spatial expansion, Urban settlements

## Abstract

Flood risk reduction at the local scale requires knowledge of the settlements which are most exposed to floods, and those where the existing measures are insufficient to handle the threats. The knowledge on spatial dynamics of the flooded human settlements is limited, especially that of the smaller ones, such as the settlements in the sub-Saharan Africa. The dataset on 122 flooded settlements in the Dosso Region (Niger) offers information on: the built-up area and the number of buildings with corrugated iron roofs in 2004, 2012, and 2019 (average dates), the type of human settlements (city, rural town, village, or hamlet), the flood dates and the number of buildings collapsed between 2011 and 2019. The data on the built-up area and the number of buildings with corrugated iron roofs were extracted by visual photointerpretation from very high-resolution images accessible through Google Earth Pro. The information on the settlement category was obtained from the Human Settlements National Directory (French acronym, ReNaLoc) published by the National Institute of Statistics of Niger. The dates of floods and the data on the number of collapsed buildings were obtained from the open access national database on flooding, known by the French acronym, BDINA. These data can be reused to build a geodatabase for flood risk reduction and to draft the municipal and regional development plans. Their potential reuse allows for the identification of settlements undergoing the most rapid physical expansion, built-up area in a flood-prone zone, and settlements that require protection and flood risk reduction policies. Additionally, the dataset can also be used to verify the accuracy of the built-up area obtained from the satellite images with coarse resolution and for comparisons with other regions in Niger and in sub-Saharan Africa.

## Specifications Table

**Table:** 

Subject	Social Sciences
Specific subject area	Planning and Development
Type of data	Tables Figures Vector files
How data were acquired	Visual photointerpretation of multi temporal very high-resolution (VHR) satellite images
Data format	Vector files in .kmz format and data tables in csv format
Parameters for data collection	The database includes only flooded settlements covered by three open access, VHR satellite images. The built-up area includes the developed lots and the roads providing access to these lots. Vacant lots, the ones under construction, and built-up lots more than 60 metres away from the contiguous built-up area are excluded. The roof material is considered only in the settlements covered by at least three clear images.
Description of data collection	All the primary data (BDINA, ReNaLoc, and VHR satellite images) are open access. The secondary data on the built-up surfaces and the buildings with corrugated iron roofs were collected by operators experienced in the observed context through visual photointerpretation of VHR satellite images. The Google Earth (GE) Pro-tools are used for built-up area tracking and surface calculation. 365 files of the built-up perimeters are collected in .kmz format and then processed in the QGIS environment for map production. For each of the 122 settlements considered, three perimeters of the built-up area are available at different dates, except for one settlement, which has two perimeters, having merged with the settlement close to the last date. The built-up surfaces are recorded on Excel.
Data source location	Primary data sources: BDINA: https://www.inondations-niger.org/ Institution: Institute of Bio Economy, National Research Council of Italy City: Florence Country: Italy ReNaLoc: https://www.stat-niger.org/?page_id=2394 Institution: Institut National de la Statistique du Niger City: Niamey, Country: Niger Satellite imagery: GE Pro Institution: Google City: Mountain View State: California, USA and Institution: Maxar Technologies City: Westminster State: Colorado, USA
Data accessibility	Repository name: Mendeley Data Data identification number: DOI: 10.17632/2xhfxdtxj2.4 Direct URL to data: https://data.mendeley.com/datasets/2xhfxdtxj2/4
Related research article	M. Tiepolo, A. Galligari, Urban expansion-flood damage nexus: Evidence from the Dosso Region, Niger, Land Use Pol. 108 (2021) 105547. https://doi.org/10.1016/j.landusepol.2021.105547.

## Value of the Data

• The built-up expansion and consolidation dataset presents an unprecedented overview of the cities, rural towns, villages, and hamlets damaged by floods between 2011 and 2019 in the Dosso Region. The accuracy of the built-up area is higher than those provided by coarse resolution satellite imagery. The dataset is useful for targeting settlements with risk reduction policies and for identifying the expansion areas.
• Local governments (municipalities), the regional government, Official development aid, NGOs, and researchers are the main beneficiaries of the data.
• The data can be used (i) to identify the most rapidly expanding settlements [1], (ii) to verify the accuracy of the built-up area obtained from the coarse resolution images, (iii) to identify the extent of the built-up area under the risk of flooding; (iv) to establish the risk reduction measures; and (v) to determine the extent and the speed of consolidation of the buildings with corrugated iron roofs.
• The value of these data lies: (i) in their detail, as they are extracted by visual photointerpretation of VHR satellite images by operators experienced with the observed context; and (ii) in their high representativeness, as they cover 40% of the flooded settlements in the Dosso region between 2011 and 2019.

## 1 Data Description

**Fig. 1:**
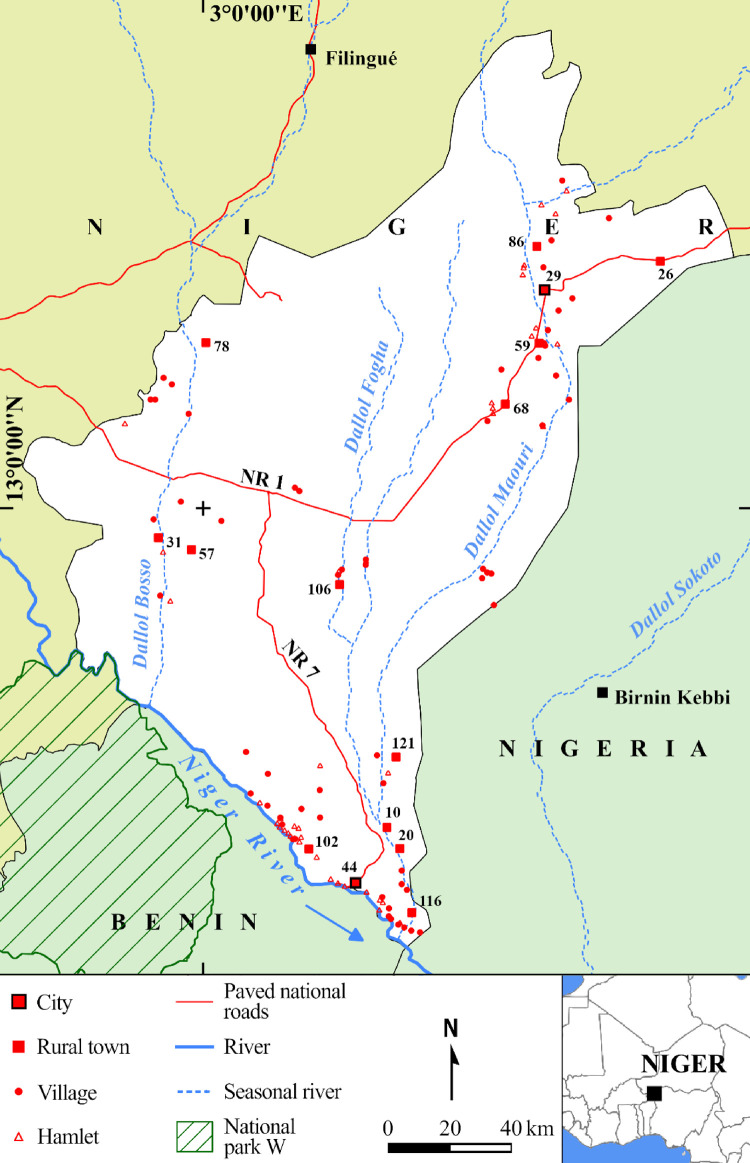
The one hundred and twenty-two flooded settlements examined in the Dosso Region based on the BDINA flood database. Figure modified from [1] showing the numbering of cities and rural towns in accordance with Table 1.

The dataset includes five figures and three tables. Fig. 1 shows the location of the 122 human settlements inundated between 2011 and 2019, covered by very high-resolution (VHR) satellite images on three dates between 2001 and 2020, accessible via GE Pro.

**Fig. 2:**
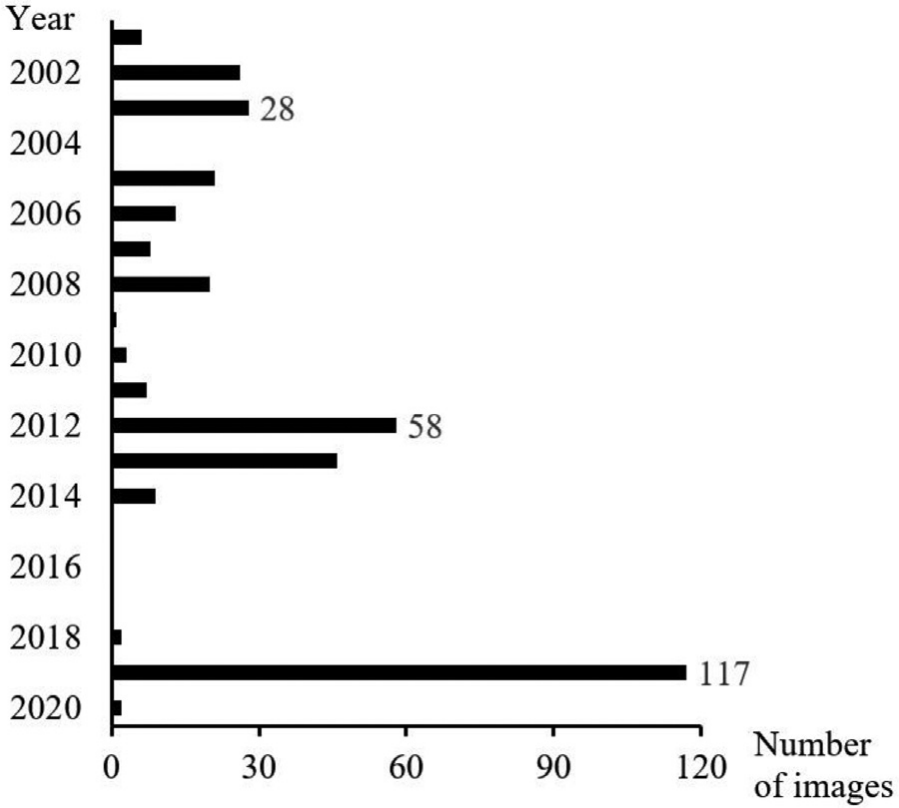
Number of VHR satellite images per year covering the flooded settlements in the Dosso Region. Figure modified from [1].

Fig. 2 shows the dates on which the VHR satellite images of the 122 settlements are freely available on GE Pro.

**Fig. 3:**
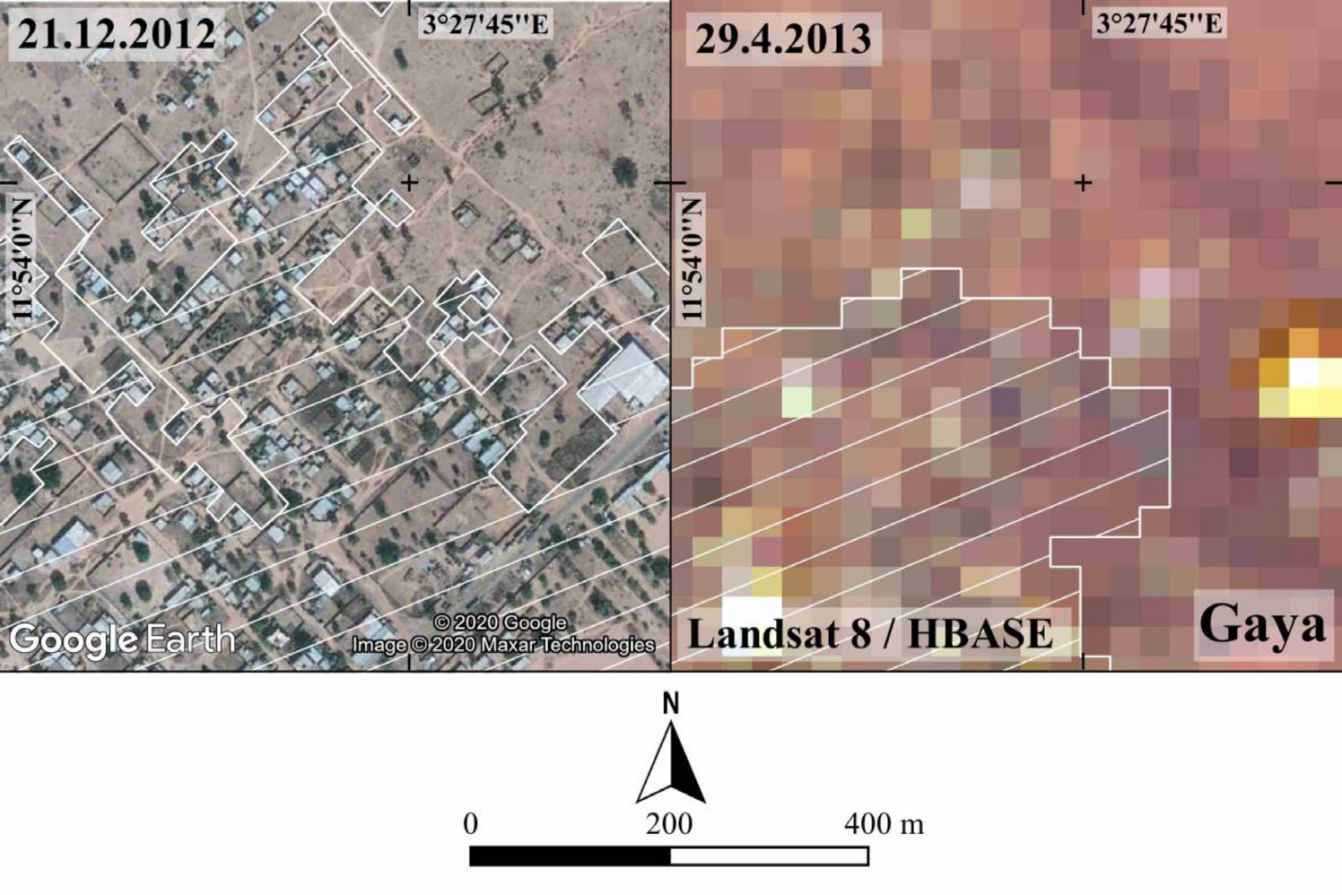
Built-up edge at Gaya city according to visual photointerpretation of GE Pro-images and according to Landsat images, respectively at 2012 and 2013 (Image©2020MaxanTechnologies).

Fig. 3 shows the accuracy in determining the built-up edge achievable through the visual photointerpretation of VHR satellite images freely accessible from GE Pro-compared to that achieved by HBASE dataset, based on Landsat 8 coarse resolution satellite images. The case illustrated is the outskirts of the city of Gaya.

**Fig. 4:**
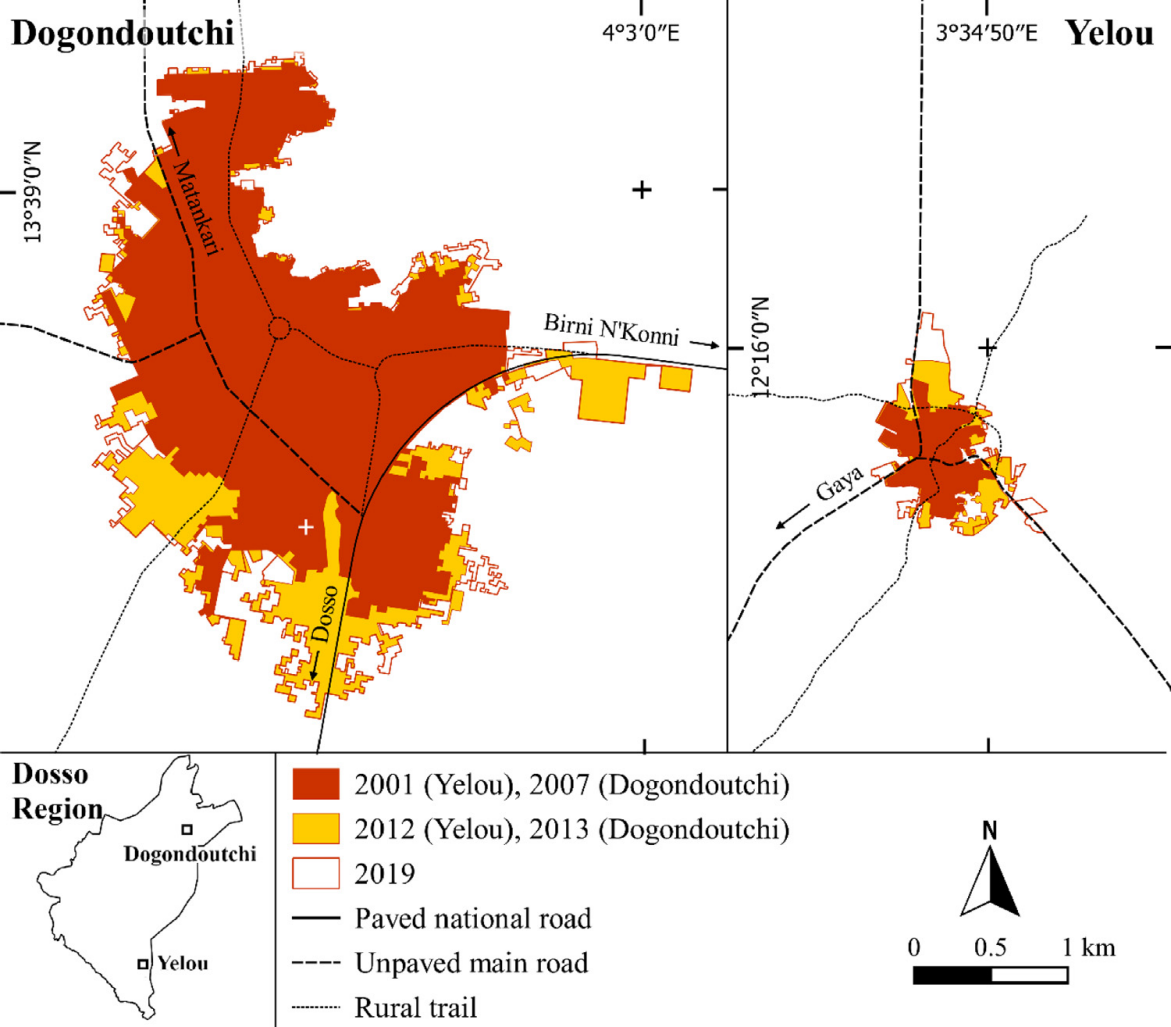
Built-up area dynamics of Dogondoutchi city between 2007 and 2019 (left) and Yelou rural town between 2001 and 2019 (right).

**Fig. 5:**
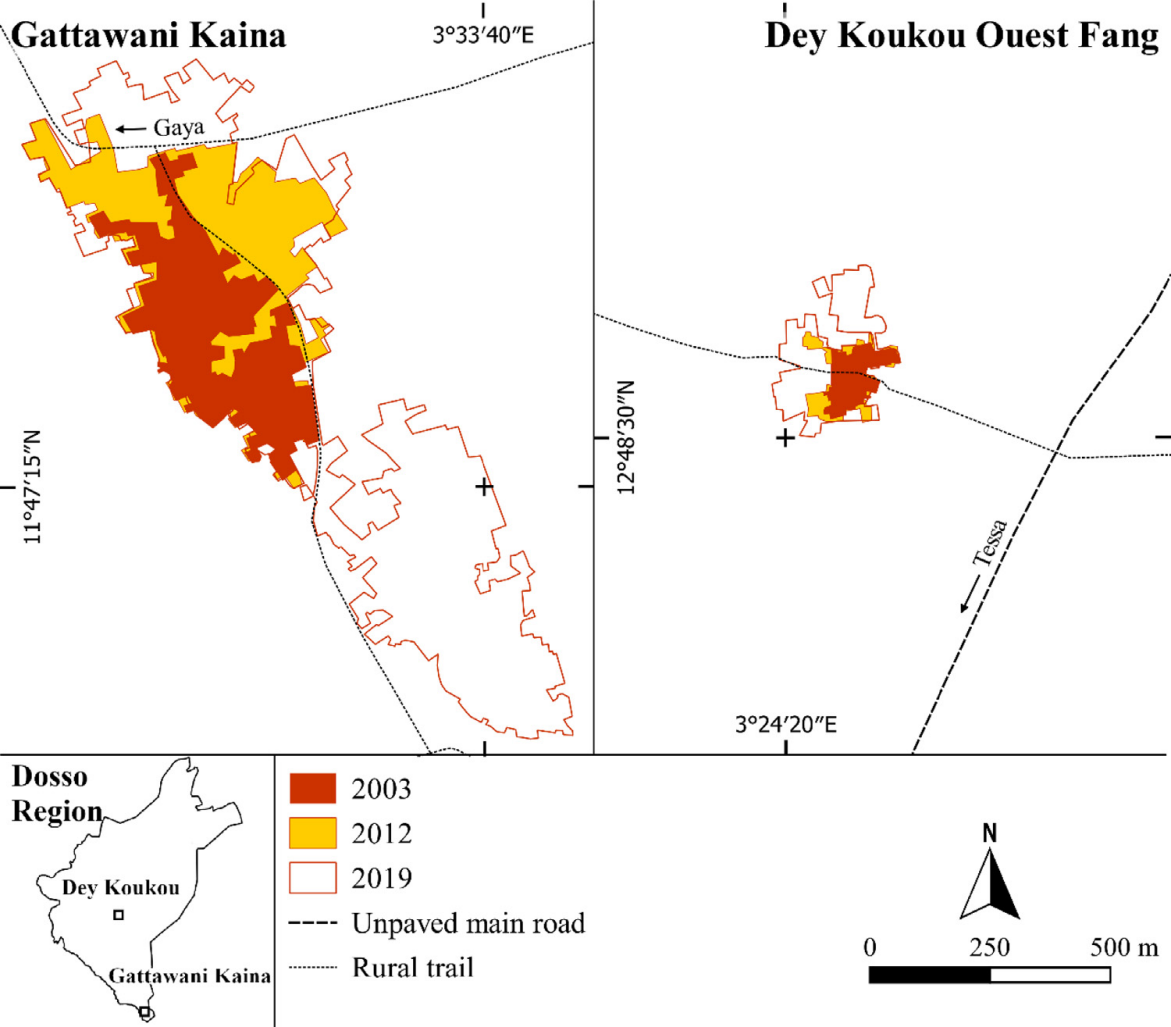
Built-up area dynamics of the Gattawani Kaina village in the municipality of Tounouga and the Dey Koukou Ouest Fang hamlet in the municipality of Tessa between 2003 and 2019.

Figs. 4 and 5 show the built-up area on the three dates for the city of Dogondoutchi, the rural town of Yelou, the village of Gattawani Kaina, and the hamlet of Dey Koukou Ouest Fang. These settlements are just four examples of some of the most rapidly expanding human settlements amongst the 122 considered. The maps are produced from shape files freely available in the Mendeley repository.

The tables list each of the 122 flooded settlements, ranked alphabetically, provided with a code that allows them to be identified in Fig. 1, the municipality and the settlement category (hamlet, village, rural town, or city) to which they belong. This is followed by data on built-up area expressed in square kilometres, the year being referred (Table 1), the number of buildings with corrugated iron roofs on three dates (the years are the same as those reported in (Table 1), and the total number of buildings in 2012. The last two fields of information are limited to only 49 settlements (Table 2).

**Table 1:** Built-up area of 122 flooded settlements in the Dosso Region in Niger based on visual photointerpretation of GE Pro-images (Image©Maxan Technologies).

		Coordinates							

#	Human settlement	Latitude N	Longitude E	Municipality	Settlement category	1st date Km^2^	2nd date Km^2^	3rd date Km^2^	VHR images year
1	Adiga Lele	12°14′4.4″	3°20′58.1″	Yelou	H	0.135	0.196	0.250	2002–13–19
2	Adoua	13°40′55.5″	3°57′48.7″	Dogondoutchi	H	0.038	0.046	0.057	2006–13–19
3	Albarkaize	12°4′57.1″	3°13′47.9″	Tanda	V	0.040	0.045	0.077	2002–13–19
4	Alfa Koara II	12°16′35.5″	3°7′37.7″	Sambera	V	0.012	0.020	0.023	2001–12–19
5	Alsandeye	12°47′56.3″	3°24′15.6″	Tessa	V	0.053	0.099	0.117	2002–13–19
6	Angoual Bozari	12°48′57.0″	3°50′26.5″	Guecheme	V	0.097	0.135	0.148	2005–13–19
7	Angoual Sani	12°15′58.3″	3°31′13.8″	Yelou	V	0.045	0.091	0.104	2001–12–19
8	Baitounga	11°48′37.1″	3°31′31.3″	Gays	H	0.002	0.002	0.003	2003–11–19
9	Bakin Tapki	13°23′8.9″	4°3′46.4″	Kieche	V	0.126	0.144	0.197	2006–13–19
10	Bana	12°3′11.5″	3°32′59.7″	Bana	RT	0.447	0.479	0.619	2010–12–19
11	Banikoubey	12°11′1.9″	3°32′22.3″	Yelou	V	0.261	0.225	0.243	2001–12–19
12	Banizoumbou Issa	13°17′18.4″	3°47′46.7″	Kore Mairoua	V	0.011	0.01	0.015	2005–13–19
13	Banizoumbou Madargu	11°45′27.9″	3°36′3.5″	Tounouga	V	0.040	0.045	0.047	2008–12–19
14	Bantali	12°3′10.6″	3°13′43.0″	Tanda	H	0.013	0.013	0.006	2006–10–19
15	Bare Bari	13°47′5.7″	4°3′2.7″	Matankari	V	0.098	0.15	0.176	2002–13–19
16	Bargoumawa	13°14′29.9″	4°1′44.5″	Tibiri	V	0.036	0.047	0.050	2003–14–19
17	Bawada	12°47′18.1″	3°50′15.5″	Guecheme	V	0.235	0.275	0.310	2005–13–19
18	Bawada Guida	13°50′58.8″	4°13′29.7″	Dan Kassari	V	0.062	0.076	0.104	2007–13–19
19	Belande Djerma	12°44′17.2″	2°52′8.3″	Falmey	V	0.368	0.439	0.430	2008–12–19
20	Bengou	11°59′26.9″	3°35′17.7″	Bengou	RT	1.090	1.160	1.330	2003–08–19
21	Biraye Garin Mallam	13°30′3.4″	3°59′23.5″	Kieche	H	0.038	0.050	0.065	2003–13–19
22	Boukka 3	13°28′37.1″	4°4′2.0″	Kieche	H	0.006	0.008	0.010	2006–13–19
23	Bouma Bamanzo	12°4′57.6″	3°20′57.0″	Tanda	V	0.006	0.010	0.022	2002–13–19
24	Boune-Boune	11°48′51.4″	3°33′20.0″	Tounouga	V	0.052	0.059	0.061	2003–11–19
25	Dadin Kowa	13°15′8.3″	3°51′19.3″	Kore Mairoua	V	0.013	0.020	0.021	2005–14–19
26	Dan Kassari	13°43′16.9″	4°22′45.8″	Dan Kassari	RT	0.646	0.791	1.200	2009–13–19
27	Darfou Tounga	12°1′11.1″	3°16′18.0″	Tanda	V	0.092	0.117	0.130	2002–12–19
28	Dey Koukou Ouest Fand	12°48′32.2″	3°24′26.0″	Tessa	H	0.013	0.019	0.048	2003–12–19
29	Dogondoutchi	13°38′18.6″	4°1′45.1″	Dogondoutchi	C	4.880	6.100	6.590	2007–13–19
30	Donoudibi Djerma	13°5′46.2″	2°55′13.0″	Birni N'Gaoure	V	0.052	0.062	0.053	2008–12–19
31	Fabidji	12°54′33.4″	2°51′48.2″	Fabidji	RT	0.456	0.682	0.815	2008–12–19
32	Foma Tounga	12°7′4.2″	3°11′28.8″	Sambera	V	0.041	0.064	0.068	2006–13–19
33	Foo	11°53′56.9″	3°22′50.8″	Gaya	H	0.017	0.023	0.033	2002–12–19
34	Gagila Mai Fala	13°26′16.4″	4°0′35.3″	Kieche	V	0.136	0.155	0.215	2006–13–19
35	Gaouna	13°53′18.1″	4°1′14.8″	Matankari	H	0.054	0.067	0.099	2007–13–19
36	Garanga	13°51′42.2″	4°3′48.9″	Matankari	H	0.008	0.010	0.011	2007–13–19
37	Garin Bana	13°43′2.3″	3°58′8.9″	Matankari	H	0.040	0.047	0.061	2005–13–19
38	Garin Dan Bina	13°24′15.4″	3°53′52.4″	Kore Mairoua	V	0.020	0.027	0.027	2003–13–19
39	Garin Kada	13°42′9.5″	3°57′58.6″	Matankari	H	0.009	0.010	0.011	2005–13–19
40	Garin Lela	12°48′18.4″	3°51′10.6″	Guecheme	V	0.027	0.034	0.052	2005–13–19
41	Garin Moudi	13°42′37.6″	3°58′5.2″	Matankari	H	0.010	0.012	0.015	2005–13–19
42	Gatawani Beri	11°46′59.0″	3°33′40.5″	Tounouga	V	0.132	0.150	0.177	2003–12–19
43	Gattawani Kaina	11°47′26.8″	3°33′17.3″	Tounouga	V	0.119	0.225	0.295	2003–12–19
44	Gaya	11°53′23.0″	3°27′19.3″	Gaya	C	3.061	5.607	6.710	2003–12–19
45	Gazere Koira	12°12′43.3″	3°11′33.4″	Sambera	V	0.017	0.020	0.023	2005–13–19
46	Goberi Goubey	12°57′48.0″	2°50′56.9″	Fabidji	V	0.152	0.172	0.230	2008–12–19
47	Gondarou	11°44′35.2″	3°38′54.4″	Tounouga	V	0.130	0.142	0.165	2008–13–19
48	Goron Kondo	11°55′32.2″	3°35′38.2″	Tounouga	V	0.060	0.084	0.105	2003–12–19
49	Gouala	13°28′26.9″	4°1′49.4″	Kieche	V	0.162	0.232	0.263	2006–13–19
50	Haoua Hanga	12°1′22.8″	3°17′31.0″	Tanda	H	0.017	0.022	0.023	2002–13–19
51	Here Damtche Peulh	12°48′6.8″	3°51′55.6″	Guecheme	V	0.063	0.088	0.114	2005–13–19
52	Himadey	12°50′39.9″	3°29′17.2″	Karguibangou	V	0.045	0.059	0.089	2005–12–19
53	Illela Makera	12°12′45.9″	3°33′13.7″	Yelou	H	0.004	0.006	0.016	2001–12–19
54	Jikata	13°14′2.2″	4°1′23.3″	Tibiri	H	0.021	0.021	0.032	2003–14–19
55	Jikata Toudou	13°13′41.9″	4°1′53.6″	Tibiri	V	0.063	0.077	0.086	2003–14–19
56	Kanaré	13°14′38.7″	2°45′44.3″	Ngonga	H	0.205	0.238	0.297	2010–11–19
57	Kankandi	12°52′25.8″	2°57′46.7″	Kankandi	RT	0.051	0.078	0.089	2002–12–19
58	Karra	13°0′56.1″	2°55′52.1″	Birni N'Gaoure	V	1.341	1.840	2.790	2002–12–19
59	Kiéché	13°28′54.0″	4°0′44.0″	Kieche	RT	0.295	0.367	0.416	2006–13–19
60	Kiota Mayaki	13°17′27.6″	2°57′18.5″	Kiota	V	1.130	1.520	1.910	2005–12–19
61	Kiota Oumarou	13°16′29.6″	2°57′15.3″	Kiota	V	0.153	0.162	0.184	2008–12–19
62	Kobassi	11°51′39.3″	3°29′17.7″	Gaya	H	0.002	0.005	0.005	2003–12–19
63	Kofo	11°50′14.2″	3°31′41.2″	Gaya	H	0.027	0.078	0.110	2003–11–19
64	Koma	11°44′53.8″	3°37′19.3″	Tounouga	V	0.038	0.042	0.051	2008–12–19
65	Komaguindi Zou	13°22′51.9″	2°52′43.8″	Harikanassou	V	0.033	0.039	0.051	2008–12–19
66	Kombo	11°52′40.6″	3°25′22.3″	Gaya	H	0.003	0.003	0.003	2002–12–19
67	Konko Rindo	13°28′54.4″	4°1′35.6″	Kieche	V	0.034	0.049	0.066	2006–12–19
68	Kore Mairoua	13°18′6.9″	3°54′34.1″	Kore Mairoua	RT	0.878	1.190	1.460	2005–13–19
69	Korwa	12°7′30.5″	3°10′6.2″	Tanda	H	0.006	0.012	0.011	2006–13–19
70	Kotcha (Tandarou)	11°53′14.8″	3°24′8.5″	Gaya	H	0.026	0.051	0.067	2002–12–19
71	Kouka Bakoye	13°34′39.1″	4°4′16.1″	Dogondoutchi	V	0.201	0.280	0.326	2006–12–19
72	Kouka Mailamba	11°57′50.5″	3°20′20.0″	Tanda	H	0.007	0.009	0.009	2002–12–20
73	Koukadin	13°55′43.3″	4°5′49.6″	Matankari	H	0.012	0.016	0.020	2007–13–19
74	Koukoki	13°16′23.3″	3°52′19.0″	Kore Mairoua	H	0.091	0.153	0.160	2005–14–19
75	Kountou Dey	13°2′48.6″	3°17′17.3″	Dosso	V	0.026	0.039	0.061	2003–12–20
76	Kourbeye	12°2′6.8″	3°15′12.6″	Tanda	H	0.010	0.019	0.022	2002–12–19
77	Kouringuel Mayaki	13°21′43.5″	2°54′13.6″	Harikanassou	V	0.175	0.181	0.232	2008–12–19
78	Koygolo	13°29′7.1″	3°0′23.3″	Koygolo	RT	0.670	0.743	0.776	2008–12–20
79	Ladan Koira	12°9′49.7″	3°20′53.5″	Tanda	V	0.046	0.074	0.078	2002–13–19
80	Lette	12°9′14.2″	3°8′26.6″	Tanda	V	0.095	0.205	0.241	2002–12–19
81	Liguido	13°36′48.4″	4°6′47.2″	Dogondoutchi	V	0.336	0.428	0.492	2007–13–19
82	Loma	13°18′51.8″	4°6′5.4″	Kore Mairoua	V	0.228	0.288	0.314	2003–13–19
83	Mabatounga	12°2′36.0″	3°14′33.0″	Tanda	H	0.006	0.01	0.015	2002–12–19
84	Magangama	12°3′50.2″	3°13′14.0″	Tanda	H	0.003	0.003	0.002	2003–12–19
85	Makera II	13°57′40.7″	4°5′2.9″	Matankari	V	0.061	0.092	0.105	2007–13–19
86	Matankari	13°46′1.7″	4°0′23.0″	Matankari	RT	1.106	1.227	1.548	2002–13–19
87	Mayaki Dey	12°48′53.8″	3°24′57.5″	Tessa	V	0.047	0.071	0.098	2005–12–19
88	Mombeye Tounga	12°1′7.4″	3°16′3.2″	Tanda	V	0.068	0.070	0.082	2002–12–19
89	Nantougou	12°1′36.1″	3°15′36.8″	Tanda	H	0.015	0.016	0.016	2002–13–19
90	Niabere Bella	13°19′1.4″	2°50′22.7″	Harikanassou	V	0.105	0.129	0.163	2008–12–19
91	Niabere Kaina	13°19′0.8″	2°51′12.9″	Harikanassou	V	0.084	0.094	0.107	2008–12–19
92	Noufawa	13°31′30.1″	4°0′11.2″	Kieche	H	0.046	0.057	0.065	2006–13–19
93	Rouda Adoua	13°31′11.9″	4°2′19.5″	Kieche	V	0.076	0.083	0.096	2006–13–19
94	Rountoua Tanda	12°1′11.0″	3°16′27.4″	Tanda	V	0.023	0.029	0.03	2002–13–18
95	Sabara Dey	12°50′19.3″	3°29′18.3″	Karguibangou	H	0.004	0.007	0.010	2005–12–19
96	Sabon Birni	11°53′8.8″	3°35′37.2″	Tounouga	V	0.607	0.794	0.917	2008–12–19
97	Sakoira	12°42′32.0″	3°52′22.1″	Guecheme	V	0.020	0.021	0.039	2005–13–19
98	Sakondji Birni	11°50′51.5″	3°32′6.5″	Gaya	V	0.022	0.022	0.028	2003–12–19
99	Sandi Tounga	11°49′52.4″	3°30′28.5″	Gaya	V	0.002	0.002	0.002	2003–12–19
100	Sia	12°6′30.2″	3°17′35.7″	Tanda	V	0.286	0.409	0.424	2002–13–19
101	Sira Lelesso	12°51′54.0″	2°52′39.0″	Fabidji	H	0.011	0.016	0.021	2008–12–19
102	Tanda	11°59′23.7″	3°18′55.4″	Tanda	RT	0.792	1.26	1.70	2002–12–19
103	Taramna	13°14′19.8″	4°1′16.1″	Tibiri	V	0.020	0.028	0.038	2003–14–19
104	Tchelele	12°0′33.4″	3°17′13.4″	Tanda	H	0.019	0.022	0.028	2002–11–20
105	Tchiara Koira	12°57′31.4″	3°3′12.0″	Gaya	V	0.054	0.088	0.089	2001–11–14
106	Tessa	12°46′14.0″	3°24′32.6″	Tessa	RT	0.385	0.505	0.556	2005–12–19
107	Togone	13°42′19.0″	4°1′34.3″	Dogondoutchi	V	0.332	0.380	0.502	2007–13–19
108	Tombo Kirey	13°3′24.1″	3°16′28.6″	Dosso	V	0.148	0.225	0.291	2003–12–19
109	Toudou	13°17′18.6″	3°52′17.5″	Kore Mairoua	H	0.010	0.013	0.016	2005–13–19
110	Toullo Maadi I	13°5′43.8″	4°5′29.9″	Tibiri	V	0.070	0.070	0.077	2003–14–19
111	Tounga Djado	11°52′7.9″	3°36′33.0″	Tounouga	V	0.010	0.011	0.012	2008–12–19
112	Tounga Goumbi	12°3′44.3″	3°14′8.0″	Tanda	V	0.024	0.047	0.043	2002–13–19
113	Tounga Maikada	11°48′28.8″	3°31′34.9″	Gaya	H	0.002	0.002	0.004	2003–11–19
114	Tounga Nadania	11°45′58.4″	3°34′59.8″	Tounouga	V	0.027	0.030	0.037	2003–12–19
115	Tounga Zaoure	11°46′12.2″	3°35′17.2″	Tounouga	H	0.018	0.018	0.023	2003–12–19
116	Tounouga	11°48′4.7″	3°37′24.4″	Tounouga	RT	0.717	0.736	0.926	2002–12–19
117	Tsaourin Boubou	13°18′14.4″	3°52′6.1″	Kore Mairoua	H	0.006	0.013	0.021	2005–13–19
118	Wadata	11°49′48.2″	3°32′13.0″	Gaya	H	0.037	0.054	0.074	2003–11–19
119	Wadata	12°4′41.6″	3°13′49.3″	Tanda	H	0.006	0.009	0.013	2002–13–19
120	Windi Bago Peulh	12°43′13.3″	2°53′57.9″	Guilladjie	H	0.025	0.034	0.046	2008–12–19
121	Yelou	12°15′39.4″	3°34′40.1″	Yelou	RT	0.350	0.539	0.668	2001–12–19
122	Zanga Babadey	12°49′42.4″	3°29′14.6″	Karguibangou	V	0.066	0.085	0.142	2003–12–19
	Total					25.700	34.280	41.560	
	Average								2004–12–19

*Abbreviations:* C-City, RT-Rural town, H-Hamlet, V-Village.

**Table 2:** Constructions (number) with corrugated iron roofs in 49 settlements of the Dosso Region, flooded between 2011 and 2019 based on visual photointerpretation of GE Pro-images (Image©Maxan Technologies).

	Number of constructions with			
	corrugated iron roof			
Human settlement	1st date	2nd date	3rd date	Number of constructions in 2012
Alsandeye	0	22	33	73
Angoual Sani	0	2	8	202
Bana	187	192	351	341
Bare Bari	10	17	42	131
Bawada Guida	8	15	26	101
Belande Djerma	52	79	233	142
Bengou	134	828	1469	1403
Bouma Bamanzo	0	0	3	52
Boune-Boune	15	42	48	49
Dan Kassari	145	260	189	761
Dey Koukou Ouest Fand	4	11	26	30
Fabidji	201	294	372	520
Gaouna	5	5	7	91
Garanga	0	0	1	6
Gattawani Kaina	17	63	201	224
Gaya	1640	6897	8216	7929
Gazere Koira	0	4	6	24
Goberi Goubey	67	73	114	172
Goron Kondo	12	64	70	115
Himadey	9	22	30	131
Kankandi	19	24	80	125
Karra	38	149	297	301
Kiota Mayaki	192	210	471	1764
Kofo	4	42	92	79
Koma	14	34	38	87
Kore Mairoua	252	398	529	1393
Koukadin	0	0	1	25
Koukoki	8	22	52	131
Kountou Dey	7	18	46	75
Koygolo	135	123	144	855
Lette	2	38	85	275
Liguido	39	95	173	631
Loma	3	25	87	226
Makera II	1	5	0	136
Matankari	254	467	627	1380
Sabara Dey	3	5	5	8
Sabon Birni	2	3	38	34
Taramna	0	6	20	76
Tessa	27	86	157	300
Togone	69	85	145	632
Tombo Kirey	30	87	225	249
Toudou	1	1	15	18
Tounga Nadania	14	25	54	69
Tounga Zaoure	6	10	28	50
Tsaourin Boubou	0	2	5	27
Wadata	5	35	66	57
Windi Bago Peulh	2	3	2	9
Yelou	33	86	253	501
Zanga Babadey	18	39	54	106
Dosso region	3684	11,013	15,234	22,116

The last table shows the number of buildings that collapsed as a result of flooding in the period 2011–19 in each of the 122 human settlements considered, as reported in the BDINA database (Table 3).

**Table 3:** Houses collapsed between 2011 and 2019 in 122 flooded settlements of the Dosso Region (Niger) according the BDINA open access database.

		Houses collapsed									
Human settlement	Category	2011	2012	2013	2014	2015	2016	2017	2018	2019	2011–19
Adiga Lele	H						96				96
Adoua	H					57					57
Albarkaize	V			96		63			250		409
Alfa Koara II	V			6							6
Alsandeye	V						4				4
Angoual Bozari	V					70					70
Angoual Sani	V								11		11
Baitounga	H			18					81		99
Bakin Tapki	V					18					18
Bana	RT	61									61
Banikoubey	V								19		19
Banizoumbou Issa	V									35	35
Banizoumbou Madarguwyo	V					11				57	68
Bantali	H					71			75		146
Bare Bari	V			16							16
Bargoumawa	V								39		39
Bawada	V					43					43
Bawada Guida	V					67					67
Belande Djerma	V								67		67
Bengou	RT	49	40								89
Biraye Garin Mallam	H	1					37				37
Boukka 3	H					6					6
Bouma Bamanzo	V								15		15
Boune	V					76				15	91
Dadin Kowa	V									3	3
Dan Kassari	RT					40				73	113
Darfou Tounga	V								125		125
Dey Koukou Ouest Fand	H						11				11
Dogondoutchi	C			540		58			14		612
Donoudibi Djerma	V					11					11
Fabidji	RT			56						50	106
Foma Tounga	V			20							20
Foo	H			10		5			60		75
Gagila Mai Fala	V		40								40
Gaouna	H			17							17
Garanga	H			27							27
Garin Bana	H								8		8
Garin Dan Bina	V								4		4
Garin Kada	H								13		13
Garin Lela	V			6		18					24
Garin Moudi	H								6		6
Gatawani Beri	V					56				42	98
Gatawani Kaina	V					68					68
Gaya	C			76				155	457	77	765
Gazere Koira	V			50							50
Goberi Goubey	V			14							14
Gondarou	V					35					35
Goron Kondo	V					15				20	35
Gouala	V									40	40
Haoua Hanga	H								98		98
Here Damtche Peulh	V			2		28					30
Himadey	V			24							24
Illela Makera	H						19				19
Jikata	H								19		19
Jikata Toudou	V								30		30
Kanaré	H			76							76
Kankandji	RT		20								20
Karra	V						128				128
Kiéché	RT					62					62
Kiota Mayaki	V						1				1
Kiota Oumarou	V			36							36
Kobassi	H			3							3
Kofo	H								65		65
Koma	V					17					17
Komaguindi Zou	V									5	5
Kombo	H			10					23		23
Konko Rindo	V					35					35
Kore Mairoua	RT						48				48
Korwa	H								80		80
Kotcha (Tandarou)	H			3		13			105		121
Kouka Bakoye	V						23				23
Kouka Mailamba	H	3									3
Koukadin	H			17							17
Koukoki	H									48	48
Kountou Dey	V								12		12
Kourbeye	H					74			114		188
Kouringuel Mayaki	V									42	42
Koygolo	RT									98	98
Ladan Koira	V						5				5
Lette	V					194			119		313
Liguido	V						74				74
Loma	V								26		26
Mabatounga	H								12		12
Magangama	H			7							7
Makera II	V			20							20
Matankari	RT			69		147			52		268
Mayaki Dei	V						27				27
Momboye Tounga	V			36		4		63	146		249
Nantougou	H								13		13
Niabere Bella	V									19	19
Niabere Kaina	V	1									1
Noufawa	H					31					31
Rouda Adoua	V					54					54
Rountoua Tanda	V			34		2			88		124
Sabara Dey	H				4						4
Sabon Birni	V					56				2	58
Sakoira	V						37				37
Sakondji Birni	V					30					30
Sandi Tounga	V										49
Sia	V								136		136
Sira Lelesso	H			11							11
Tanda	RT								14		14
Taramna	V	32									32
Tchelele	H					15			34		49
Tchiara Koira	V										22
Tessa	RT						22				22
Togone	V								35		35
Tombo Kirey	V					82			28		16
Toudou	H							17			17
Toullou Maadi I	V								10		10
Tounga Djado	V					10					10
Tounga Goumbi	V			56				168	173		397
Tounga Maikada	H			24						14	38
Tounga Nadania	V			9							9
Tounga Zaoure	H										10
Tounouga	RT					67					67
Tsaourin Boubou	H									8	8
Wadata	H								30		30
Wadata	H								4		4
Windi Bago Peulh	H			65							65
Yelou	RT								44		44
Zanga Babadey	V			24							24

*Abbreviations:* C-City, RT-Rural town, H-Hamlet, V-Village.

## 2 Experimental Design, Materials and Methods

The flooded settlements in the Dosso Region were obtained from the open access national database on flooding-BDINA (www.inondations-niger.org) [2]. This source of information provides access to data on flooded sites, loss and damage as collected annually since 1998 by the Early Warning System Coordination Unit through its network of observers on the ground and currently run by the Ministry of Humanitarian Action and Disaster Management. This source has proved to be much richer and more up to date than the global flood databases as Desinventar, the Dartmouth observatory on floods, the EM-DAT, and is therefore used here [3]. Further, 290 flooded settlements were determined between 2011 and 2019 from this primary dataset. These settlements were then identified on satellite images through the geographical coordinates listed in the Human Settlements National Directory, ReNaLoc [4]. For each flooded settlement, the availability of at least three VHR images on GE Pro-were verified over the course of the last 20 years. Therefore, 122 settlements (Fig. 1) were documented by the images captured between 2001 and 2009 (2004 on average), 2010 and 2014 (2012 on average), 2018 and 2020 (2019 on average) (Fig. 2).

Settlements were characterised into urban and rural according to the definition provided by the National Institute of Statistics of Niger. The first category includes cities, i.e. all regional or departmental capitals (41,000 inhabitants on average). The rural category includes the capitals of rural municipalities (5300 inhabitants on average), villages, the lowest level at which taxes are collected and health and education services are provided (1200 inhabitants on average), and hamlets, simple aggregations of dwellings without administrative functions and community services (500 inhabitants on average) [5,6].

The built-up area of each settlement was visually identified on three dates including all the contiguous developed lots and the road surfaces providing access to these lots. Playgrounds, graveyards, and communication tower lots are also included. Vacant lots, the ones under construction, and the isolated developed lots are excluded when they are separated from the contiguous built-up area more than 60 m away or two standard vacant lots [7] (Fig. 3).

The built-up area is acquired using the “View” and the “Historic imagery” buttons of GE Pro. It was then manually measured using the “Add polygon” GE Pro-tool. A .kmz file is then generated for each built-up area in each year and for the settlement, thereby generating a total of 365 files, freely available at the Mendeley repository. These files are transferred into the QGIS environment to initiate the map production (Figs. 4 and 5).

In rural Niger, adobe buildings prevail. Substituting the earthen roof with a corrugated iron roof is the most common measure to protect houses from heavy rains. Corrugated iron sheets are identified through the visual photointerpretation of VHR satellite images, which are accessible with true colours from GE Pro. The images captured in the months of February-March (two-thirds) and September (22%) were not usable. Conversely, the images captured in December-January and June-August have suitable illumination and excellent atmospheric conditions and allowed for the coverage of 40% of the settlements with three images within the period of interest. The colours light grey or light blue indicate corrugated iron roofs. However, as the region is seasonally exposed to dust winds the colour of the roof can be masked. To limit visual photointerpretation errors field inspections were conducted in the villages of Gagila, Sabon Birni, and Takouidawa and in the rural towns of Guéchémé and Tessa (Fig. 3). However, only images captured from unmanned aerial vehicles show without any doubt the roof material [8].

The effectiveness of roof retrofitting can be appreciated by comparing the widespread use of corrugated iron sheet in building roofs with the number of buildings that have collapsed as a result of pluvial flooding between 2011 and 2019. The latter were taken from the BDINA open access database for the human settlements considered (Table 3). In a few cases flood damage is recorded by the BDINA for clusters of human settlements (three to ten villages and hamlets). To overcame this lack of detail, the number of collapsed buildings for the settlement of interest was estimated by multiplying the number of collapsed buildings in the cluster by the population of the settlement of interest and dividing the result by the total population of the cluster.

## Ethics Statement

No ethical issues are associated with this study.

## Data Availability

Data on Flooded Settlements in the Dosso Region, Niger (Original data) (Mendeley Data).

## CRediT authorship contribution statement

**Maurizio Tiepolo:** Conceptualization, Methodology, Software, Data curation, Writing – original draft. **Andrea Galligari:** Software, Visualization, Writing – review & editing.

## Declaration of Competing Interest

The authors declare that they have no known competing financial interests or personal relationships which have or could be perceived to have influenced the work reported in this article.

## Appendix Supplementary materials

Image, application 1
