# Supplementary material for: Dataset on the expansion and consolidation of flooded settlements in the Dosso Region, Niger
Source: Data Brief. 2022 Feb 10;41:107935. doi: 10.1016/j.dib.2022.107935 (PMC8857557; doi:10.1016/j.dib.2022.107935)
Supplement: Supplementary file 1 [file mmc1.docx]

**Table A.** Peer-reviewed literature on urban expansion in sub-Saharan Africa, 2011-2020.

| **Human settlement** | **Population** | **Built-up area** | **Reference** |
| --- | --- | --- | --- |

| **Country**  **ISO 3** | **City/town** | **> 1 M** | **1-0.5 M** | **0.5-0.1 M** | **< 0.1 M** | **Start-End** | **Observed years** | **ha** | **Satellite images** | **Author** |
| --- | --- | --- | --- | --- | --- | --- | --- | --- | --- | --- |
| BWA | Gabane |  |  |  | 1 | 1982-2012 | 30 | 73 | unk | Mpofu 2018 |
| CAM | Bamenda |  |  | 1 |  | 1989-2015 | 26 | 1,986 | Landsat | Mbanga 2020 |
| CAM | Tubah |  |  |  | 1 | 1983-2013 | 30 | 3,105 | Landsat | Nguh 2017 |
| CDI | Abidjan | 1 |  |  |  | 1990-2016 | 26 | 20,140 | Landsat | Abdelaziz 2020 |
| CDI | Abobo | 1 |  |  |  | 1987-2019 | 32 | 4,768 | Landsat | Koné 2020 |
| COD | Kinshasa | 1 |  |  |  | 1979-2015 | 36 | 53,556 | Landsat | Messina Ndzomo 2019 |
| COD | Lumumbashi | 1 |  |  |  | 1989-2014 | 25 | 32,240 | SPOT | Sikuzani 2018 |
| COG | Brazzaville | 1 |  |  |  | 2001-2011 | 10 | 3,819 | Landsat | Kempena 2014 |
| CPV | Praia |  |  |  | 1 | 1969-2015 | 46 | 1,028 | Landsat+Maps | Silva 2017 |
| ERI | Asmara |  | 1 |  |  | 1989-2009 | 20 | 5,905 | Landsat | Tewolde 2011 |
| ETH | Adama |  |  | 1 |  | 1984-2015 | 31 | 2,275 | Landsat | Sinha 2016 |
| ETH | Addis | 1 |  |  |  | 1986-2011 | 25 | 20,900 | Landsat | Arsisio 2018 |
| ETH | Axum |  |  |  | 1 | 1985-2015 | 30 | 719 | Landsat | Gebre Medhin 2019 |
| ETH | Bahir Dar |  |  | 1 |  | 1957-2009 | 52 | 4,830 | ERDAS | Haregeweyn 2012 |
| ETH | Bahir Dar |  |  |  |  | 1973-2015 | 42 | 2,740 | Landsat | Gashu 2018 |
| ETH | Burayu |  |  |  | 1 | 1987-2019 | 32 | 3,234 | Landsat | Terfa 2020 |
| ETH | Debre Tabor |  |  |  | 1 | 1997-2017 | 20 | 1,670 | Landsat | Halefom 2018 |
| ETH | Dukem |  |  |  | 1 | 1987-2019 | 32 | 2,138 | Landsat | Terfa 2020 |
| ETH | Dire Dawa |  |  | 1 |  | 1985-2016 | 31 | 4,466 | UNK | Erena 2019 |
| ETH | Gelan |  |  |  | 1 | 1987-2019 | 32 | 1,220 | Landsat | Terfa 2020 |
| ETH | Gondar |  |  | 1 |  | 2004-2017 | 13 | 5,303 | Landsat | Jothimani 2019 |
| ETH | Hawassa |  |  | 1 |  | 1973-2015 | 42 | 1,261 | Landsat | Gashu 2018 |
| ETH | Hawassa |  |  |  |  | 1973-2006 | 33 | 4,044 | GE | Admasu 2015 |
| ETH | Jigjiga |  |  | 1 |  | 1985-2015 | 30 | 1,795 | Landsat | Barow 2019 |
| ETH | Jimma |  |  | 1 |  | 1973-2019 | 46 | 4,436 | Landsat | Hailu 2020 |
| ETH | Laga Tafo |  |  |  | 1 | 1996-2016 | 20 | 953 | Landsat-SPOT | Kebebew 2019 |
| ETH | Lege-Tafo |  |  |  |  | 1987-2019 | 32 | 1,888 | Landsat | Terfa 2020 |
| ETH | Mekelle |  |  | 1 |  | 1984-2014 | 30 | 3,524 | Landsat | Fenta 2017 |
| ETH | Nekemte |  |  | 1 |  | 1996-2016 | 20 | 1,322 | GE | Megersa 2018 |
| ETH | Sebeta |  |  | 1 |  | 1987-2019 | 32 | 3,450 | Landsat | Terfa 2020 |
| ETH | Sabeta |  |  |  |  | 2003-2016 | 13 | 3,799 | Landsat | Sabeta 2019 |
| ETH | Sululta |  |  |  | 1 | 1987-2019 | 32 | 1,206 | Landsat | Tafu 2020 |
| GHA | Accra |  | 1 |  |  | 1991-2015 | 24 | 70,355 | Landsat | Addae 2019 |
| GHA | Kumasi |  | 1 |  |  | 1986-2016 | 30 | 22,610 | Landsat | Abass 2018 |
| GHA | New Juabeng |  |  |  | 1 | 1985-2015 | 30 | 4,963 | Landsat | Nyamekye 2020 |
| GHA | Sekondi |  |  |  | 1 | 1986-2016 | 30 | 9,089 | Landsat | Acheampong 2018 |
| GHA | Tema |  |  | 1 |  | 1990-2007 | 17 | 15,000 | Landsat | Mariwah 2017 |
| GHA | Wa |  |  | 1 |  | 1986-2016 | 30 | 3,293 | Landsat | Korah 2018 |
| GIN | Pita |  |  |  | 1 | 1985-2015 | 30 | 5,871 | Landsat | Cissé 2020 |
| KEN | Morogoro |  |  | 1 |  | 2000-2016 | 16 | 4,333 | Landsat | Sumari 2020 |
| KEN | Nairobi |  |  |  |  | 1976-2000 | 24 | 6,125 | Landsat | Mundia 2010 |
| KEN | Nairobi |  |  |  |  | 1999-2014 | 15 | 9,230 | Landsat | Hao 2016 |
| KEN | Nairobi |  | 1 |  |  | 1995-2015 | 20 | 349,462 | Landsat | Katjambo 2017 |
| KEN | Ruaka Town |  |  |  | 1 | 1988-2019 | 31 | 747 | Landsat | Abuya 2019 |
| KEN | Thika |  |  | 1 |  | 1976-2010 | 34 | no | Landsat | Muiuri 2017 |
| MAL | Bamako |  | 1 |  |  | 1986-2006 | 20 | 10,248 | Landsat | Diallo 2010 |
| MAL | Bamako |  |  |  |  | 1999-2014 | 15 | 12,290 | Landsat | Hao 2016 |
| MWI | Blantyre |  | 1 |  |  | 1994-2018 | 24 | 4,393 | Landsat | Mawenda 2020 |
| MOZ | Beira |  | 1 |  |  | 1998-2018 | 20 | 8,275 | Landsat | Barbosa 2019 |
| NIG | Abuja |  |  |  |  | 1986-2014 | 28 | 1,310 | Landsat | Mahmoud 2016 |
| NIG | Abuja | 1 |  |  |  | 1987-2017 | 30 | 38,540 | Landsat | Enoguanbhor 2019 |
| NIG | Akure |  |  | 1 |  | 1972-2002 | 30 | 3,853 | Landsat | Eke 2017 |
| NIG | Akure |  |  | 1 |  | 1985-2015 | 30 | 57,629 | Landsat | Yakubu 2020 |
| NIG | Awka |  |  |  | 1 | 1986-2016 | 30 | 11,452 | Landsat | Chunwate 2019 |
| NIG | Benin City | 1 |  |  |  | 2002-2013 | 11 | 35,900 | Landsat | Odjugo 2015 |
| NIG | Benin Metro |  |  |  |  | 1989-2013 | 24 | 42,531 | Landsat | Nkeki 2016 |
| NIG | Damaturu |  |  |  | 1 | 1987-2017 | 30 | 1,906 | Landsat | Babagana-Kyari 2020 |
| NIG | Gombe |  |  | 1 |  | 1976-2016 | 40 | 3,615 | Landsat | Mbaya 2019 |
| NIG | Ibadan | 1 |  |  |  | 1984-2019 | 35 | 52,085 | Landsat | Fashae 2020 |
| NIG | Ife |  | 1 |  |  | 1986-2009 | 23 | 6,694 | Landsat | Oloukoi 2014 |
| NIG | Ilorin | 1 |  |  |  | 1986-2010 | 24 | 49,863 | Landsat | Suleiman 2014 |
| NIG | Jos |  | 1 |  |  | 1984-2014 | 30 | 6,194 | Landsat | Bello 2019 |
| NIG | Jos |  |  |  |  | 1984-2014 | 30 | 2,654 | Landsat | Akintunde 2016 |
| NIG | Kaduna | 1 |  |  |  | 1987-2016 | 29 | 3,509 | Landsat | Ezeamaka 2019 |
| NIG | Kano | 1 |  |  |  | 1986-2005 | 19 | 9,018 | Landsat | Ayila 2014 |
| NIG | Lagos | 1 |  |  |  | 1984-2013 | 29 | 23,048 | Landsat | Babalola 2016 |
| NIG | Maiduguiri | 1 |  |  |  | 2000-2018 | 18 | 9,631 | Landsat | Akintunde |
| NIG | Makurdi |  |  | 1 |  | 1986-2016 | 30 | 2,922 | Landsat | Acha 2018 |
| NIG | Ogbomoso | 1 |  |  |  | 1984-2016 | 32 | 86,460 | Landsat | Adeoye 2018 |
| NIG | Otukpo |  |  | 1 |  | 1987-2017 | 30 | 15,475 | Landsat | Jande 2020 |
| NIG | Owerri | 1 |  |  |  | 1986-2016 | 30 | 55,297 | Landsat | Ukaegbu 2017 |
| NIG | Port Harcourt | 1 |  |  |  | 1986-2003 | 17 | 55,000 | Landsat | Dan-Jumbo 2018 |
| NIG | Sokoto |  | 1 |  |  | 1986-2016 | 30 | 3,170 | Landsat | Ogunjobi 2018 |
| NIG | Ukwuani |  |  |  | 1 | 1990-2014 | 24 | 2,269 | Landsat | Wizor 2020 |
| NIG | Uyo |  |  | 1 |  | 1986-2017 | 31 | 10,793 | Landsat | Essien 2019 |
| NIG | Warri |  | 1 |  |  | 1987-2007 | 20 | 23,676 | Landsat | Gobo 2014 |
| NIG | Zaria | 1 |  |  |  | 1990-2009 | 19 | 28,900 | Landsat | Aminu 2013 |
| CAF | Bangui |  |  | 1 |  | 1986-2020 | 34 | 8,680 | Landsat | Traore 2020 |
| REU | Saint Denis |  |  | 1 |  | 2008-2013 | 5 | 690 | SPOT Pléiades | Thibault 2015 |
| RWA | Kigali |  | 1 |  |  | 1984-2016 | 32 | 10,017 | Landsat | Mugiraneza 2019 |
| SLE | Bo |  |  |  | 1 | 2000-2015 | 15 | 2,899 | Landsat | Tarawally 2019 |
| SLE | Freetown | 1 |  |  |  | 2000-2015 | 15 | 12,832 | Landsat | Tarawally 2019 |
| TZA | Dar es Salaam | 1 |  |  |  | 2002-2011 | 9 | 37,688 | Landsat | Congedo 2014 |
| TZA | Dodoma |  |  | 1 |  | 1998-2018 | 20 | 2,793 | Landsat | Kabanda 2019 |
| TZA | Morogoro |  |  |  | 1 | 2000-2016 | 16 | 4,333 | Landsat | Sumari 2019 |
| TZA | Zanzibar |  |  |  | 1 | 2003-2018 | 15 | 7,201 | Landsat | Cabral 2020 |
| UGA | Mbarara |  |  |  | 1 | 1984-2014 | 30 | 648 | Landsat | Bwanika 2016 |
| ZAF | Port Elizabeth |  | 1 |  |  | 1990-2000 | 10 | 18,900 | Landsat | Odindi 2013 |
| ZAF | Tshwane |  | 1 |  |  | 1984-2015 | 31 | 168,475 | Landsat | Magidi 2019 |
| ZMB | Lusaka |  | 1 |  |  | 1990-2010 | 20 | 15,836 | Landsat | Simwanda 2017 |
| ZMB | Lusaka |  |  |  |  | 1995-2015 | 20 | 28,411 | Landsat | Nguvulu 2017 |
| ZWE | Harare |  | 1 |  |  | 1984-2013 | 29 | 34,220 | Landsat | Kamusoko 2013 |
| ZWE | Harare |  |  |  |  | 1984-2018 | 34 | 54,497 | Landsat | Marondedze 2019 |
| Tot./Avg. |  | 20 | 16 | 23 | 21 |  | 26 |  |  |  |

**Table B.** Built-up area of 124 flooded settlements in the Dosso Region, Niger according to visual photointerpretation of Google Earth images (Image©Maxan Technologies).

Abbreviations: C-City, CRM-Capital town of rural municipality, H-Hamlet, V-Village.

| **Human settlement** | **Coordinates** | | **Municipality** | **Settlement category** | **1^st^ date** | **2^nd^ date** | **3^rd^ date** | **1^st^ image** | **2^nd^ image** | **3^rd^ image** |
| --- | --- | --- | --- | --- | --- | --- | --- | --- | --- | --- |
|  | Latitude (N) | Longitude (E) |  |  | ha | ha | ha | year | year | year |
| Adiga Lele | 12°14′4.4″ | 3°20′58.1″ | Yelou | H | 13.5 | 19.6 | 25 | 2002 | 2013 | 2019 |
| Adoua | 13°40′55.5″ | 3°57′48.7″ | Dogondoutchi | H | 3.8 | 4.6 | 5.7 | 2006 | 2013 | 2019 |
| Albarkaize | 12°4′57.1″ | 3°13′47.9″ | Tanda | V | 4 | 4.5 | 7.7 | 2002 | 2013 | 2019 |
| Alfa Koara II | 12°16′35.5″ | 3°7′37.7″ | Sambera | V | 1.2 | 2 | 2.3 | 2001 | 2012 | 2019 |
| Alsandeye | 12°47′56.3″ | 3°24′15.6″ | Tessa | V | 5.3 | 9.9 | 11.7 | 2002 | 2013 | 2019 |
| Angoual Bozari | 12°48′57.0″ | 3°50′26.5″ | Guecheme | V | 9.7 | 13.5 | 14.8 | 2005 | 2013 | 2019 |
| Angoual Sani | 12°15′58.3″ | 3°31′13.8″ | Yelou | V | 4.5 | 9.1 | 10.4 | 2001 | 2012 | 2019 |
| Baitounga | 11°48′37.1″ | 3°31′31.3″ | Gays | H | 0.2 | 0.2 | 0.3 | 2003 | 2011 | 2019 |
| Bakin Tapki | 13°23′8.9″ | 4°3′46.4″ | Kieche | V | 12.6 | 14.4 | 19.7 | 2006 | 2013 | 2019 |
| Bana | 12°3′11.5″ | 3°32′59.7″ | Bana | CRM | 44.7 | 47.9 | 61.9 | 2010 | 2012 | 2019 |
| Banikoubey | 12°11′1.9″ | 3°32′22.3″ | Yelou | V | 26.1 | 22.5 | 24.3 | 2001 | 2012 | 2019 |
| Banizoumbou Issa | 13°17′18.4″ | 3°47′46.7″ | Kore Mairoua | V | 1.1 | 1 | 1.5 | 2005 | 2013 | 2019 |
| Banizoumbou Madargu | 11°45′27.9″ | 3°36′3.5″ | Tounouga | V | 4 | 4.5 | 4.7 | 2008 | 2012 | 2019 |
| Bantali | 12°3′10.6″ | 3°13′43.0″ | Tanda | H | 1.3 | 1.3 | 0.6 | 2006 | 2010 | 2019 |
| Bare Bari | 13°47′5.7″ | 4°3′2.7″ | Matankari | V | 9.8 | 15 | 17.6 | 2002 | 2013 | 2019 |
| Bargoumawa | 13°14′29.9″ | 4°1′44.5″ | Tibiri | V | 3.6 | 4.7 | 5 | 2003 | 2014 | 2019 |
| Bawada | 12°47′18.1″ | 3°50′15.5″ | Guecheme | V | 23.5 | 27.5 | 31 | 2005 | 2013 | 2019 |
| Bawada Guida | 13°50′58.8″ | 4°13′29.7″ | Dan Kassari | V | 6.2 | 7.6 | 10.4 | 2007 | 2013 | 2019 |
| Belande Djerma | 12°44′17.2″ | 2°52′8.3″ | Falmey | V | 36.8 | 43.9 | 43 | 2008 | 2012 | 2019 |
| Bengou | 11°59′26.9″ | 3°35′17.7″ | Bengou | CRM | 109 | 116 | 133 | 2003 | 2008 | 2019 |
| Biraye Garin Mallam | 13°30′3.4″ | 3°59′23.5″ | Kieche | H | 3.8 | 5 | 6.5 | 2003 | 2013 | 2019 |
| Boukka 3 | 13°28′37.1″ | 4°4′2.0″ | Kieche | H | 0.6 | 0.8 | 1 | 2006 | 2013 | 2019 |
| Bouma Bamanzo | 12°4′57.6″ | 3°20′57.0″ | Tanda | V | 0.6 | 1 | 2.2 | 2002 | 2013 | 2019 |
| Boune-Boune | 11°48′51.4″ | 3°33′20.0″ | Tounouga | V | 5.2 | 5.9 | 6.1 | 2003 | 2011 | 2019 |
| Dadin Kowa | 13°15′8.3″ | 3°51′19.3″ | Kore Mairoua | V | 1.3 | 2 | 2.1 | 2005 | 2014 | 2019 |
| Dan Kassari | 13°43′16.9″ | 4°22′45.8″ | Dan Kassari | CRM | 64.6 | 79.1 | 120 | 2009 | 2013 | 2019 |
| Darfou Tounga | 12°1′11.1″ | 3°16′18.0″ | Tanda | V | 9.2 | 11.7 | 13 | 2002 | 2012 | 2019 |
| Dey Koukou Ouest Fand | 12°48′32.2″ | 3°24′26.0″ | Tessa | H | 1.3 | 1.9 | 4.8 | 2003 | 2012 | 2019 |
| Dogondoutchi | 13°38′18.6″ | 4°1′45.1″ | Dogondoutchi | C | 488 | 610 | 659 | 2007 | 2013 | 2019 |
| Donoudibi Djerma | 13°5′46.2″ | 2°55′13.0″ | Birni N'Gaoure | V | 5.2 | 6.2 | 5.3 | 2008 | 2012 | 2019 |
| Fabidji | 12°54′33.4″ | 2°51′48.2″ | Fabidji | CRM | 45.6 | 68.2 | 81.5 | 2008 | 2012 | 2019 |
| Foma Tounga | 12°7′4.2″ | 3°11′28.8″ | Sambera | V | 4.1 | 6.4 | 6.8 | 2006 | 2013 | 2019 |
| Foo | 11°53′56.9″ | 3°22′50.8″ | Gaya | H | 1.7 | 2.3 | 3.3 | 2002 | 2012 | 2019 |
| Gagila Mai Fala | 13°26′16.4″ | 4°0′35.3″ | Kieche | V | 13.6 | 15.5 | 21.5 | 2006 | 2013 | 2019 |
| Gaouna | 13°53′18.1″ | 4°1′14.8″ | Matankari | H | 5.4 | 6.7 | 9.9 | 2007 | 2013 | 2019 |
| Garanga | 13°51′42.2″ | 4°3′48.9″ | Matankari | H | 0.8 | 1 | 1.1 | 2007 | 2013 | 2019 |
| Garin Bana | 13°43′2.3″ | 3°58′8.9″ | Matankari | H | 4 | 4.7 | 6.1 | 2005 | 2013 | 2019 |
| Garin Dan Bina | 13°24′15.4″ | 3°53′52.4″ | Kore Mairoua | V | 2 | 2.7 | 2.7 | 2003 | 2013 | 2019 |
| Garin Kada | 13°42′9.5″ | 3°57′58.6″ | Matankari | H | 0.9 | 1 | 1.1 | 2005 | 2013 | 2019 |
| Garin Lela | 12°48′18.4″ | 3°51′10.6″ | Guecheme | V | 2.7 | 3.4 | 5.2 | 2005 | 2013 | 2019 |
| Garin Moudi | 13°42′37.6″ | 3°58′5.2″ | Matankari | H | 1 | 1.2 | 1.5 | 2005 | 2013 | 2019 |
| Gatawani Beri | 11°46′59.0″ | 3°33′40.5″ | Tounouga | V | 13.2 | 15 | 17.7 | 2003 | 2012 | 2019 |
| Gattawani Kaina | 11°47′26.8″ | 3°33′17.3″ | Tounouga | V | 11.9 | 22.5 | 29.5 | 2003 | 2012 | 2019 |
| Gaya | 11°53′23.0″ | 3°27′19.3″ | Gaya | C | 306.1 | 560.7 | 671 | 2003 | 2012 | 2019 |
| Gazere Koira | 12°12′43.3″ | 3°11′33.4″ | Sambera | V | 1.7 | 2 | 2.3 | 2005 | 2013 | 2019 |
| Goberi Goubey | 12°57′48.0″ | 2°50′56.9″ | Fabidji | V | 15.2 | 17.2 | 23 | 2008 | 2012 | 2019 |
| Gondarou | 11°44′35.2″ | 3°38′54.4″ | Tounouga | V | 13 | 14.2 | 16.5 | 2008 | 2013 | 2019 |
| Goron Kondo | 11°55′32.2″ | 3°35′38.2″ | Tounouga | V | 6 | 8.4 | 10.5 | 2003 | 2012 | 2019 |
| Gouala | 13°28′26.9″ | 4°1′49.4″ | Kieche | V | 16.2 | 23.2 | 26.3 | 2006 | 2013 | 2019 |
| Haoua Hanga | 12°1′22.8″ | 3°17′31.0″ | Tanda | H | 1.7 | 2.2 | 2.3 | 2002 | 2013 | 2019 |
| Here Damtche Peulh | 12°48′6.8″ | 3°51′55.6″ | Guecheme | V | 6.3 | 8.8 | 11.4 | 2005 | 2013 | 2019 |
| Himadey | 12°50′39.9″ | 3°29′17.2″ | Karguibangou | V | 4.5 | 5.9 | 8.9 | 2005 | 2012 | 2019 |
| Illela Makera | 12°12′45.9″ | 3°33′13.7″ | Yelou | H | 0.4 | 0.6 | 1.6 | 2001 | 2012 | 2019 |
| Jikata | 13°14′2.2″ | 4°1′23.3″ | Tibiri | H | 2.1 | 2.1 | 3.2 | 2003 | 2014 | 2019 |
| Jikata Toudou | 13°13′41.9″ | 4°1′53.6″ | Tibiri | V | 6.3 | 7.7 | 8.6 | 2003 | 2014 | 2019 |
| Kanaré | 13°14′38.7″ | 2°45′44.3″ | Ngonga | H | 20.5 | 23.8 | 29.7 | 2010 | 2011 | 2019 |
| Kankandi | 12°52′25.8″ | 2°57′46.7″ | Kankandi | CRM | 5.1 | 7.8 | 8.9 | 2002 | 2012 | 2019 |
| Karra | 13°0′56.1″ | 2°55′52.1″ | Birni N'Gaoure | V | 134.1 | 184 | 279 | 2002 | 2012 | 2019 |
| Kiéché | 13°28′54.0″ | 4°0′44.0″ | Kieche | CRM | 29.5 | 36.7 | 41.6 | 2006 | 2013 | 2019 |
| Kiota Mayaki | 13°17′27.6″ | 2°57′18.5″ | Kiota | V | 113 | 152 | 191 | 2005 | 2012 | 2019 |
| Kiota Oumarou | 13°16′29.6″ | 2°57′15.3″ | Kiota | V | 15.3 | 16.2 | 18.4 | 2008 | 2012 | 2019 |
| Kobassi | 11°51′39.3″ | 3°29′17.7″ | Gaya | H | 0.2 | 0.5 | 0.5 | 2003 | 2012 | 2019 |
| Kofo | 11°50′14.2″ | 3°31′41.2″ | Gaya | H | 2.7 | 7.8 | 11 | 2003 | 2011 | 2019 |
| Koma | 11°44′53.8″ | 3°37′19.3″ | Tounouga | V | 3.8 | 4.2 | 5.1 | 2008 | 2012 | 2019 |
| Komaguindi Zou | 13°22′51.9″ | 2°52′43.8″ | Harikanassou | V | 3.3 | 3.9 | 5.1 | 2008 | 2012 | 2019 |
| Kombo | 11°52′40.6″ | 3°25′22.3″ | Gaya | H | 0.3 | 0.3 | 0.3 | 2002 | 2012 | 2019 |
| Konko Rindo | 13°28′54.4″ | 4°1′35.6″ | Kieche | V | 3.4 | 4.9 | 6.6 | 2006 | 2012 | 2019 |
| Kore Mairoua | 13°18′6.9″ | 3°54′34.1″ | Kore Mairoua | CRM | 87.8 | 119 | 146 | 2005 | 2013 | 2019 |
| Korwa | 12°7′30.5″ | 3°10′6.2″ | Tanda | H | 0.6 | 1.2 | 1.1 | 2006 | 2013 | 2019 |
| Kotcha (Tandarou) | 11°53′14.8″ | 3°24′8.5″ | Gaya | H | 2.6 | 5.1 | 6.7 | 2002 | 2012 | 2019 |
| Kouka Bakoye | 13°34′39.1″ | 4°4′16.1″ | Dogondoutchi | V | 20.1 | 28 | 32.6 | 2006 | 2012 | 2019 |
| Kouka Mailamba | 11°57′50.5″ | 3°20′20.0″ | Tanda | H | 0.7 | 0.9 | 0.9 | 2002 | 2012 | 2020 |
| Koukadin | 13°55′43.3″ | 4°5′49.6″ | Matankari | H | 1.2 | 1.6 | 2 | 2007 | 2013 | 2019 |
| Koukoki | 13°16′23.3″ | 3°52′19.0″ | Kore Mairoua | H | 9.1 | 15.3 | 16 | 2005 | 2014 | 2019 |
| Kountou Dey | 13°2′48.6″ | 3°17′17.3″ | Dosso | V | 2.6 | 3.9 | 6.1 | 2003 | 2012 | 2020 |
| Kourbeye | 12°2′6.8″ | 3°15′12.6″ | Tanda | H | 1 | 1.9 | 2.2 | 2002 | 2012 | 2019 |
| Kouringuel Mayaki | 13°21′43.5″ | 2°54′13.6″ | Harikanassou | V | 17.5 | 18.1 | 23.2 | 2008 | 2012 | 2019 |
| Koygolo | 13°29′7.1″ | 3°0′23.3″ | Koygolo | CRM | 67 | 74.3 | 77.6 | 2008 | 2012 | 2020 |
| Ladan Koira | 12°9′49.7″ | 3°20′53.5″ | Tanda | V | 4.6 | 7.4 | 7.8 | 2002 | 2013 | 2019 |
| Lette | 12°9′14.2″ | 3°8′26.6″ | Tanda | V | 9.5 | 20.5 | 24.1 | 2002 | 2012 | 2019 |
| Liguido | 13°36′48.4″ | 4°6′47.2″ | Dogondoutchi | V | 33.6 | 42.8 | 49.2 | 2007 | 2013 | 2019 |
| Loma | 13°18′51.8″ | 4°6′5.4″ | Kore Mairoua | V | 22.8 | 28.8 | 31.4 | 2003 | 2013 | 2019 |
| Mabatounga | 12°2′36.0″ | 3°14′33.0″ | Tanda | H | 0.6 | 1.1 | 1.5 | 2002 | 2012 | 2019 |
| Magangama | 12°3′50.2″ | 3°13′14.0″ | Tanda | H | 0.3 | 0.3 | 0.2 | 2003 | 2012 | 2019 |
| Makera II | 13°57′40.7″ | 4°5′2.9″ | Matankari | V | 6.1 | 9.2 | 10.5 | 2007 | 2013 | 2019 |
| Matankari | 13°46′1.7″ | 4°0′23.0″ | Matankari | CRM | 110.6 | 122.7 | 154.8 | 2002 | 2013 | 2019 |
| Mayaki Dey | 12°48′53.8″ | 3°24′57.5″ | Tessa | V | 4.7 | 7.1 | 9.8 | 2005 | 2012 | 2019 |
| Mombeye Tounga | 12°1′7.4″ | 3°16′3.2″ | Tanda | V | 6.8 | 7 | 8.2 | 2002 | 2012 | 2019 |
| Nantougou | 12°1′36.1″ | 3°15′36.8″ | Tanda | H | 1.5 | 1.6 | 1.6 | 2002 | 2013 | 2019 |
| Niabere Bella | 13°19′1.4″ | 2°50′22.7″ | Harikanassou | V | 10.5 | 12.9 | 16.3 | 2008 | 2012 | 2019 |
| Niabere Kaina | 13°19′0.8″ | 2°51′12.9″ | Harikanassou | V | 8.4 | 9.4 | 10.7 | 2008 | 2012 | 2019 |
| Noufawa | 13°31′30.1″ | 4°0′11.2″ | Kieche | H | 4.6 | 5.7 | 6.5 | 2006 | 2013 | 2019 |
| Rouda Adoua | 13°31′11.9″ | 4°2′19.5″ | Kieche | V | 7.6 | 8.3 | 9.6 | 2006 | 2013 | 2019 |
| Rountoua Tanda | 12°1′11.0″ | 3°16′27.4″ | Tanda | V | 2.3 | 2.9 | 3 | 2002 | 2013 | 2018 |
| Sabara Dey | 12°50′19.3″ | 3°29′18.3″ | Karguibangou | H | 0.4 | 0.7 | 1 | 2005 | 2012 | 2019 |
| Sabon Birni | 11°53′8.8″ | 3°35′37.2″ | Tounouga | V | 60.7 | 79.4 | 91.7 | 2008 | 2012 | 2019 |
| Sakoira | 12°42′32.0″ | 3°52′22.1″ | Guecheme | V | 2 | 2.1 | 3.9 | 2005 | 2013 | 2019 |
| Sakondji Birni | 11°50′51.5″ | 3°32′6.5″ | Gaya | V | 2.2 | 2.2 | 2.8 | 2003 | 2012 | 2019 |
| Sandi Tounga | 11°49′52.4″ | 3°30′28.5″ | Gaya | V | 0.2 | 0.2 | 0.2 | 2003 | 2012 | 2019 |
| Sia | 12°6′30.2″ | 3°17′35.7″ | Tanda | V | 28.6 | 40.9 | 42.4 | 2002 | 2013 | 2019 |
| Sira Lelesso | 12°51′54.0″ | 2°52′39.0″ | Fabidji | H | 1.1 | 1.6 | 2.1 | 2008 | 2012 | 2019 |
| Tanda | 11°59′23.7″ | 3°18′55.4″ | Tanda | CRM | 79.2 | 126 | 170 | 2002 | 2012 | 2019 |
| Taramna | 13°14′19.8″ | 4°1′16.1″ | Tibiri | V | 2 | 2.8 | 3.8 | 2003 | 2014 | 2019 |
| Tchelele | 12°0′33.4″ | 3°17′13.4″ | Tanda | H | 1.9 | 2.2 | 2.8 | 2002 | 2011 | 2020 |
| Tchiara Koira | 12°57′31.4″ | 3°3′12.0″ | Gaya | V | 5.4 | 8.8 | 8.9 | 2001 | 2011 | 2014 |
| Tessa | 12°46′14.0″ | 3°24′32.6″ | Tessa | CRM | 38.5 | 50.5 | 55.6 | 2005 | 2012 | 2019 |
| Togone | 13°42′19.0″ | 4°1′34.3″ | Dogondoutchi | V | 33.2 | 38 | 50.2 | 2007 | 2013 | 2019 |
| Tombo Kirey | 13°3′24.1″ | 3°16′28.6″ | Dosso | V | 14.8 | 22.5 | 29.1 | 2003 | 2012 | 2019 |
| Toudou | 13°17′18.6″ | 3°52′17.5″ | Kore Mairoua | H | 1 | 1.3 | 1.6 | 2005 | 2013 | 2019 |
| Toullo Maadi I | 13°5′43.8″ | 4°5′29.9″ | Tibiri | V | 7 | 7 | 7.7 | 2003 | 2014 | 2019 |
| Tounga Djado | 11°52′7.9″ | 3°36′33.0″ | Tounouga | V | 1 | 1.1 | 1.2 | 2008 | 2012 | 2019 |
| Tounga Goumbi | 12°3′44.3″ | 3°14′8.0″ | Tanda | V | 2.4 | 4.7 | 4.3 | 2002 | 2013 | 2019 |
| Tounga Maikada | 11°48′28.8″ | 3°31′34.9″ | Gaya | H | 0.2 | 0.2 | 0.4 | 2003 | 2011 | 2019 |
| Tounga Nadania | 11°45′58.4″ | 3°34′59.8″ | Tounouga | V | 2.7 | 3 | 3.7 | 2003 | 2012 | 2019 |
| Tounga Zaoure | 11°46′12.2″ | 3°35′17.2″ | Tounouga | H | 1.8 | 1.8 | 2.3 | 2003 | 2012 | 2019 |
| Tounouga | 11°48′4.7″ | 3°37′24.4″ | Tounouga | CRM | 71.7 | 73.6 | 92.6 | 2002 | 2012 | 2019 |
| Tsaourin Boubou | 13°18′14.4″ | 3°52′6.1″ | Kore Mairoua | H | 0.6 | 1.3 | 2.1 | 2005 | 2013 | 2019 |
| Wadata | 11°49′48.2″ | 3°32′13.0″ | Gaya | H | 3.7 | 5.4 | 7.4 | 2003 | 2011 | 2019 |
| Wadata | 12°4′41.6″ | 3°13′49.3″ | Tanda | H | 0.6 | 0.9 | 1.3 | 2002 | 2013 | 2019 |
| Windi Bago Peulh | 12°43′13.3″ | 2°53′57.9″ | Guilladjie | H | 2.5 | 3.4 | 4.6 | 2008 | 2012 | 2019 |
| Yelou | 12°15′39.4″ | 3°34′40.1″ | Yelou | CRM | 35 | 53.9 | 66.8 | 2001 | 2012 | 2019 |
| Zanga Babadey | 12°49′42.4″ | 3°29′14.6″ | Karguibangou | V | 6.6 | 8.5 | 14.2 | 2003 | 2012 | 2019 |
| Total |  |  |  |  | 2,570 | 3,428 | 4,156 |  |  |  |
| Average |  |  |  |  |  |  |  | 2004 | 2012 | 2019 |

**Table C.** Corrugated iron roof in 50 flooded human settlements of the Dosso Region, Niger according to visual photointerpretation of Google Earth images (Image©Maxan Technologies).

Abbreviations: C-City, CRM-Capital town of rural municipality, H-Hamlet, V-Village.

| **Human settlement** | **Coordinates** |  | **Municipality** | **Category** | **Corrugated iron roof** | | | **Roof total** |
| --- | --- | --- | --- | --- | --- | --- | --- | --- |
|  | Latitude (N) | Longitude (E) |  |  | **1^st^ date** | **2^nd^ date** | **3^rd^ date** | **2012** |
|  |  |  |  |  | **n.** | **n.** | **n.** | **n.** |
| Alsandeye | 12°47′56.3″ | 3°24′15.6″ | Tessa | V | 0 | 22 | 33 | 73 |
| Angoual Sani | 12°15′58.3″ | 3°31′13.8″ | Yelou | V | 0 | 2 | 8 | 202 |
| Bana | 12°3′11.5″ | 3°32′59.7″ | Bana | CRM | 187 | 192 | 351 | 341 |
| Bare Bari | 13°47′5.7″ | 4°3′2.7″ | Matankari | V | 10 | 17 | 42 | 131 |
| Bawada Guida | 13°50′58.8″ | 4°13′29.7″ | Dan Kassari | V | 8 | 15 | 26 | 101 |
| Belande Djerma | 12°44′17.2″ | 2°52′8.3″ | Falmey | V | 52 | 79 | 233 | 142 |
| Bengou | 11°59′26.9″ | 3°35′17.7″ | Bengou | CRM | 134 | 828 | 1469 | 1,403 |
| Bouma Bamanzo | 12°4′57.6″ | 3°20′57.0″ | Tanda | V | 0 | 0 | 3 | 52 |
| Boune-Boune | 11°48′51.4″ | 3°33′20.0″ | Tounouga | V | 15 | 42 | 48 | 49 |
| Dan Kassari | 13°43′16.9″ | 4°22′45.8″ | Dan Kassari | CRM | 145 | 260 | 189 | 761 |
| Dey Koukou Ouest Fand | 12°48′32.2″ | 3°24′26.0″ | Tessa | H | 4 | 11 | 26 | 30 |
| Fabidji | 12°54′33.4″ | 2°51′48.2″ | Fabidji | CRM | 201 | 294 | 372 | 520 |
| Gaouna | 13°53′18.1″ | 4°1′14.8″ | Matankari | H | 5 | 5 | 7 | 91 |
| Garanga | 13°51′42.2″ | 4°3′48.9″ | Matankari | H | 0 | 0 | 1 | 6 |
| Gattawani Kaina | 11°47′26.8″ | 3°33′17.3″ | Tounouga | V | 17 | 63 | 201 | 224 |
| Gaya | 11°53′23.0″ | 3°27′19.3″ | Gaya | C | 1,640 | 6,897 | 8,216 | 7,929 |
| Gazere Koira | 12°12′43.3″ | 3°11′33.4″ | Sambera | V | 0 | 4 | 6 | 24 |
| Goberi Goubey | 12°57′48.0″ | 2°50′56.9″ | Fabidji | V | 67 | 73 | 114 | 172 |
| Goron Kondo | 11°55′32.2″ | 3°35′38.2″ | Tounouga | V | 12 | 64 | 70 | 115 |
| Himadey | 12°50′39.9″ | 3°29′17.2″ | Karguibangou | V | 9 | 22 | 30 | 131 |
| Kankandi | 12°52′25.8″ | 2°57′46.7″ | Kankandi | CRM | 19 | 24 | 80 | 125 |
| Karra | 13°0′56.1″ | 2°55′52.1″ | Birni N'Gaoure | V | 38 | 149 | 297 | 301 |
| Kiota Mayaki | 13°17′27.6″ | 2°57′18.5″ | Kiota | V | 192 | 210 | 471 | 1,764 |
| Kofo | 11°50′14.2″ | 3°31′41.2″ | Gaya | H | 4 | 42 | 92 | 79 |
| Koma | 11°44′53.8″ | 3°37′19.3″ | Tounouga | V | 14 | 34 | 38 | 87 |
| Kore Mairoua | 13°18′6.9″ | 3°54′34.1″ | Kore Mairoua | CRM | 252 | 398 | 529 | 1393 |
| Koukadin | 13°55′43.3″ | 4°5′49.6″ | Matankari | H | 0 | 0 | 1 | 25 |
| Koukoki | 13°16′23.3″ | 3°52′19.0″ | Kore Mairoua | H | 8 | 22 | 52 | 131 |
| Kountou Dey | 13°2′48.6″ | 3°17′17.3″ | Dosso | V | 7 | 18 | 46 | 75 |
| Koygolo | 13°29′7.1″ | 3°0′23.3″ | Koygolo | CRM | 135 | 123 | 144 | 855 |
| Lette | 12°9′14.2″ | 3°8′26.6″ | Tanda | V | 2 | 38 | 85 | 275 |
| Liguido | 13°36′48.4″ | 4°6′47.2″ | Dogondoutchi | V | 39 | 95 | 173 | 631 |
| Loma | 13°18′51.8″ | 4°6′5.4″ | Kore Mairoua | V | 3 | 25 | 87 | 226 |
| Makera II | 13°57′40.7″ | 4°5′2.9″ | Matankari | V | 1 | 5 | 0 | 136 |
| Matankari | 13°46′1.7″ | 4°0′23.0″ | Matankari | CRM | 254 | 467 | 627 | 1,380 |
| Sabara Dey | 12°50′19.3″ | 3°29′18.3″ | Karguibangou | H | 3 | 5 | 5 | 8 |
| Sabon Birni | 11°53′8.8″ | 3°35′37.2″ | Tounouga | V | 2 | 3 | 38 | 34 |
| Taramna | 13°14′19.8″ | 4°1′16.1″ | Tibiri | V | 0 | 6 | 20 | 76 |
| Tessa | 12°46′14.0″ | 3°24′32.6″ | Tessa | CRM | 27 | 86 | 157 | 300 |
| Togone | 13°42′19.0″ | 4°1′34.3″ | Dogondoutchi | V | 69 | 85 | 145 | 632 |
| Tombo Kirey | 13°3′24.1″ | 3°16′28.6″ | Dosso | V | 30 | 87 | 225 | 249 |
| Toudou | 13°17′18.6″ | 3°52′17.5″ | Kore Mairoua | H | 1 | 1 | 15 | 18 |
| Tounga Nadania | 11°45′58.4″ | 3°34′59.8″ | Tounouga | V | 14 | 25 | 54 | 69 |
| Tounga Zaoure | 11°46′12.2″ | 3°35′17.2″ | Tounouga | H | 6 | 10 | 28 | 50 |
| Tsaourin Boubou | 13°18′14.4″ | 3°52′6.1″ | Kore Mairoua | H | 0 | 2 | 5 | 27 |
| Wadata | 11°49′48.2″ | 3°32′13.0″ | Gaya | H | 5 | 35 | 66 | 57 |
| Windi Bago Peulh | 12°43′13.3″ | 2°53′57.9″ | Guilladjie | H | 2 | 3 | 2 | 9 |
| Yelou | 12°15′39.4″ | 3°34′40.1″ | Yelou | CL | 33 | 86 | 253 | 501 |
| Zanga Babadey | 12°49′42.4″ | 3°29′14.6″ | Karguibangou | V | 18 | 39 | 54 | 106 |
| Dosso region |  |  |  |  | 3,684 | 11,013 | 15,234 | 22,116 |
